# Prevalence of Congenital Anomalies Before and After Implementation of a Novel Wastewater Treatment Technology in Texas: An Augmented Synthetic Control Method Analysis Utilizing Data from a Population-Based Birth Defects Registry

**DOI:** 10.23889/ijpds.v9i5.2743

**Published:** 2024-09-18

**Authors:** Jeremy Schraw, Kara Rudolph, Charles Shumate, Matthew Gribble

**Affiliations:** 1 Baylor College of Medicine; 2 Columbia University; 3 Texas Department of State Health Services; 4 University of California San Francisco

## Objectives and Approach

Direct potable reuse (DPR) involves adding purified wastewater that has not passed through an environmental buffer into a water distribution system. This technology may address growing demand for water in many population centers, but there are no studies of health outcomes in populations receiving DPR-treated drinking water. Our objective was to determine whether the prevalence of congenital anomalies increased in the period following introduction of DPR-treated water to certain public water systems in Texas. We obtained data on all cases with congenital anomalies regardless of pregnancy outcome during 2003-2017 from the population-based Texas Birth Defects Registry. Maternal demographic and residential data were obtained and a reference population of all livebirths in Texas was identified by linkage to birth and fetal death records. The augmented synthetic control method was used to model county-level changes in prevalence of congenital anomalies (expressed per 10,000 livebirths) after the adoption of DPR by four Texas counties in mid-2013. County-level data on maternal age, education, ethnicity, and rural-urban status were included as covariates. Results: We observed increased prevalence of all congenital anomalies collectively (average treatment effect in the treated [ATT] = 53.6) and congenital heart disease (ATT = 287.3) during the years 2014-2017. Neural tube defects prevalence was unchanged.

## Conclusions

We found evidence that the prevalence of congenital anomalies overall and congenital heart disease specifically increased during the years 2014-2017.

## Implications

Additional research into reproductive and developmental outcomes among people in areas supplied with DPR-treated water is warranted to inform water policy decisions.

